# Embedded 3D Printing of Novel Bespoke Soft Dosage Form Concept for Pediatrics

**DOI:** 10.3390/pharmaceutics11120630

**Published:** 2019-11-26

**Authors:** Katarzyna Rycerz, Krzysztof Adam Stepien, Marta Czapiewska, Basel T. Arafat, Rober Habashy, Abdullah Isreb, Matthew Peak, Mohamed A. Alhnan

**Affiliations:** 1 School of Pharmacy and Biomedical Sciences, University of Central Lancashire, Preston, Lancashire PR1 2HE, UK; katarzyna.rycerz7@gmail.com (K.R.); krzysztof.stepien@wum.edu.pl (K.A.S.); marta.czapiewska@cm.umk.pl (M.C.); robo_85@hotmail.com (R.H.); AIsreb@uclan.ac.uk (A.I.); 2 Faculty of Pharmacy with the Laboratory Medicine Division, Medical University of Warsaw, 02-091 Warsaw, Poland; 3 Faculty of Pharmacy, Department of Pharmaceutical Technology, Collegium Medicum in Bydgoszcz, Nicolaus Copernicus University in Toruń, Toruń, Jurasza 2 St., 85-089 Bydgoszcz, Poland; 4 Faculty of Medical Sciences and Public Health, Anglia Ruskin University, Chelmsford CM1 1SQ, UK; Basel.arafat@anglia.ac.uk; 5 Paediatric Medicines Research Unit, Alder Hey Children’s NHS Foundation Trust, Liverpool L12 2AP, UK; Matthew.Peak@alderhey.nhs.uk; 6 Institute of Pharmaceutical Sciences, School of Cancer and Pharmaceutical Sciences, King’s College London, London SE1 9NH, UK

**Keywords:** personalized medicine, additive manufacturing, complex structures, tablets, patient-specific, structural design, gums

## Abstract

Embedded three-dimensional printing (e-3DP) is an emerging method for additive manufacturing where semi-solid materials are extruded within a solidifying liquid matrix. Here, we present the first example of employing e-3DP in the pharmaceutical field and demonstrate the fabrication of bespoke chewable dosage forms with dual drug loading for potential use in pediatrics. Lego^TM^-like chewable bricks made of edible soft material (gelatin-based matrix) were produced by directly extruding novel printing patterns of model drug ink (embedded phase) into a liquid gelatin-based matrix (embedding phase) at an elevated temperature (70 °C) to then solidify at room temperature. Dose titration of the two model drugs (paracetamol and ibuprofen) was possible by using specially designed printing patterns of the embedded phase to produce varying doses. A linearity [R^2^ = 0.9804 (paracetamol) and 0.9976 (ibuprofen)] was achieved between percentage of completion of printing patterns and achieved doses using a multi-step method. The impact of embedded phase rheological behavior, the printing speed and the needle size of the embedded phase were examined. Owning to their appearance, modular nature, ease of personalizing dose and geometry, and tailoring and potential inclusion of various materials, this new dosage form concept holds a substantial promise for novel dosage forms in pediatrics.

## 1. Introduction

With increasing regulatory incentives for pediatric formulation development and focus in delivering patient-centred health care, developing age-appropriate formulations is gaining increased interest [1] from the pharmaceutical sector. Within Europe, the pediatric regulation on improving medicines for children was amended to include a requirement to evaluate the acceptability of formulations of medicines for children seeking a marketing authorization. There have been increasing efforts to provide high-quality, effective, and safe formulations for children [2]. The use of unlicensed and off-label medicines in pediatrics is extensive [3]. A lack of age-appropriate formulations for children means that available medicines are frequently modified to achieve an intended dose and/or administration to the child [3]. For example, tablets may need to be split by healthcare professionals or families to achieve an intended dose, with the potential for significant under- or over-dosing [4,5].

Chewable jelly or jelly-like oral doses have been proposed as easy-to-handle and to swallow for the elderly population and are often prepared using gelatin, glycogelatin, and caseinate [6]. The inclusion of drug in the structure provides an opportunity for taste masking and avoids the need for drinking water [7]. Several products of chewable gels and dosage forms are commercially available, particularly for the delivery of vitamins [8], mineral supplements [9], nutrition [10], as well as for the treatment of osteoporosis [11,12]. However, formulating chewable oral doses is often associated with challenges regarding drug stability and the capacity for higher doses.

Several reports have showed the potential of 3D printing in controlling dosage [13,14], drug combination [15,16,17], and proposed modification of pediatric-friendly shapes [18,19,20,21]. However, there are limited examples of using 3D printing for fabrication of patient-specific chewable oral dosage forms. Stereolithographic 3D printing can also produce gel tablets [22]; however, developing oral dosage forms with this approach is hindered by the difficulty of acceptance by regulatory bodies of necessary polymer initiators required for 3D printing. Fused deposition modeling (FDM) 3D printing has also been used to produce child-friendly shapes and has achieved improved taste-masking [18]. In a recent report, Goyanes et al. (2019) provided personalized chewable formulations prepared by direct ink writing of pectin-based gel for the treatment of maple syrup urine disease. Such an approach requires a formulation change to accommodate each model drug.

Embedded 3D printing (e-3DP) is an emerging technology that allows the free form fabrication of multiminerals with complex structures. The method involves extruding a viscoelastic ink (embedded phase) using a deposition nozzle at a predefined path into a solidifying reservoir (embedding phase). Following printing, the reservoir is solidified usually through a curing method to form a monolithic structure. e-3DP has been used for production of highly programmable and seamless structures such as wearable electronics [23]. Applying this approach in the pharmaceutical field holds the opportunity of providing a modular system of a generic matrix that embeds one or more drugs at individualized doses to meet the need of one or a small number of patients. The aim of this work is to apply e-3DP to pharmaceutical grade material to craft bespoke oral concept chewable dosage forms for pediatrics. Lego™-like bricks made of chewable soft material were produced by applying novel printing patterns of model drug suspension (embedded phase) into a liquid gelatin-based matrix (embedding phase). We studied the impact of printing materials composition, process parameters and demonstrated the system ability of providing bespoke dosage control and dual drug release by manipulating printing patterns. The two model drugs (paracetamol and ibuprofen) are often used in combination or alternately for the treatment of febrile children [24,25,26].

## 2. Materials and Methods

### 2.1. Materials

Ibuprofen (grade 25) was donated by BASF (Burgbernheim, Germany). Paracetamol (≥99.0%) and gelatin were purchased from Sigma-Aldrich (Darmstadt, Germany). Glycerol EP was supplied by J.M. Loveridge Ltd. (Andover, UK). Locust bean gum (from *Ceratonia siliqia* seeds) were purchased from Sigma-Aldrich (Poole, UK). Food dyes (Brilliant Blue and Lake Allura Red) were supplied by FastColours LLP (Huddersfield, UK).

### 2.2. Preparation of Embedding and Embedded Materials

Each oral dosage form was composed of ibuprofen or paracetamol powder suspension in locust gum solution (paste) and embedding medium (gelatin-based gel).

#### 2.2.1. Embedding Medium

An optimized weight ratio of water: glycerol: gelatin (45:25:30) was used as a thermo-responsive embedding medium. Initially, water was heated to 75 °C followed by the addition of gelatin, with stirring, until complete dissolution.

#### 2.2.2. Embedded Drug Paste

A solution of 2.98% *w/w* locust bean (at temperature 80 °C) was used to suspend model drug particles in the medium. For paracetamol, suspensions (100 g) were prepared at different drug concentrations: 20%, 30%, 40%, 50%, or 60% in locust bean solution. For ibuprofen, suspensions (100 g) were prepared at different drug concentrations: 16%, 22%, 28%, 34%, or 40% *w/w* in locust bean solution. The suspensions were colored by adding 5 mg of Brilliant Blue (paracetamol) and Lake Allura Red (ibuprofen) and manually stirred for 2 min for homogenization. Following rheological and experimental printing (Section 2.6), the concentrations of 40% (paracetamol) and 28% (ibuprofen) were chosen as default for printing oral doses.

### 2.3. Modification of Dual FDM 3D Printer

In order to develop a process of manufacturing novel soft dosage forms via e-3DP, a MakerBot Replicator Experimental 2X dual FDM 3D printer (MakerBot Industries, Brooklyn, NY, USA) was modified as highlighted in Supplementary Data, Figure S1. The 3D printer has two FDM nozzle heads. The right extruder/head of the dual 3D printer was replaced by a syringe-based liquid dispenser. The design for the dispenser was obtained from an open-source design (Thingiverse, 2017) and the different parts were produced by 3D printing using a M2 MakerGear FDM 3D printer and acrylonitrile butadiene styrene (ABS) filaments (MakerGear LLC, Beachwood, OH, USA). The dispenser head was installed and equipped with either a 2.5 mL or a 10 mL syringe. A Nema 17 1.5 A 4-lead stepper motor (MakerBot Industries, Brooklyn, NY, USA) was connected to the motherboard using the default housing connectors [26].

### 2.4. Design of Template and Pattern

The template design was produced by 3D printing using a M2 MakerGear FDM 3D printer and ABS filaments (MakerGear LLC) and Simplify 3D software (Simplify 3D LLC, Cincinnati, OH, USA). A Lego^TM^-like design was chosen to test proof-of-concept that a complex shape and a design familiar to children could be produced using e-3DP. A Lego^TM^-like template was printed using polylactic acid (PLA) filament (MakerBot). The design of the pattern for printing the paste with active substance was prepared using Autodesk^®^ 3ds Max^®^ Design version 2018 (Autodesk Inc., San Rafael, CA, USA). The design was saved in STL format and imported to Simplify 3D software (version 4.1) (Simplify 3D LLC, Cincinnati, OH, USA). During experimentation, printing the paste with the active substance in the desired pattern was carried out using a modified MakerBot Replicator Experimental 2X and using Simplify 3D software. The dimensions for the embedded printing pattern were: X × Y × Z = 20 × 29.52 × 0.45 mm and were optimized to fit within a gelatin-based Lego™-like brick (40 × 25 × 15 mm). For 25%, 50%, and 75% printing patterns, the designs had identical X and Z dimensions whilst the Y axis was 8.1, 15, or 22.5 mm, respectively. Printing the full design (100%) corresponded to doses of approximately 107 and 115 mg for ibuprofen and paracetamol, respectively. In order to demonstrate the capacity of the system for printing lower and higher doses, the printing pattern was printed at 25%, 50%, or 75% of the full design for smaller doses and the design was repeated twice (200%) or three times (300%) to achieve larger doses. All printing patterns were printed within identical Lego™-like templates as detailed above.

### 2.5. Embedded 3D Printing Process

Two methods of embedding the printing patterns in the gelatin matrix were performed:

#### 2.5.1. One-Step Embedded 3D Printing

To embed a drug suspension pattern into warm gelatin, the embedding medium was first heated to approximately 70 °C and cast into a Lego^TM^ shape template and placed on the printing plate of the 3D printer heated to 75 °C. Parameters (positions, dimensions, printing speed, extrusion multiplier) were programmed in the Simplify3D software. Prior to printing the embedded layer(s), the building platform was heated to 75 °C and gelatin was poured into the template. The G-code was modified to allow the needle tip to start extrusion at a height of 4 mm from the bottom of the design. The extrusion multiplier was set at 10.0 and the size of the needle was G16. The printing patterns were assessed at the following needle speeds: 50, 55, 60, or 65 mm/min.

#### 2.5.2. Multi-Step Embedded 3D Printing

Embedding the drug into the structure was performed using three steps: (i) casting liquid gelatin solution (6 mL) inside the template and left to cool to solidify; (ii) printing the drug-paste (embedded phase) on the surface of the semi-solid gelatin; and (iii) covering the drug-paste with a second portion (5 mL) of liquid gelatin. In order to allow the printing of two model drugs at two separate specific levels, the printing was carried out by casting 4 mL of embedding phase followed by paracetamol printing and then an additional 3 mL of embedding phase. Following the printing of the ibuprofen layer, a final layer of gelatin (4 mL) was cast.

The following settings were used: retraction distance 1 mm, retraction speed 1200 mm, extrusion width manual 0.4 mm, outline direction: inside to outside, and movement speed *x* − *y* 500 mm/min and *z*:150 mm/min. Infill printing speeds of 45, 50, 55, or 60 mm/s and extrusion multiplier of 3×, 5×, or 10×, and luer lock needle head with different nozzle sizes of G15, G16, and G17 (McMaster Ltd., Chicago, IL, USA) were screened. Following the analysis of a range of different printing parameters, the following parameters, 55 mm/min, nozzle size G16, and extrusion multiplier 5× were chosen as default to print different paracetamol and ibuprofen doses.

### 2.6. Rheological Studies of Embedded Material

A shear Physica MCR 102 rheometer (Anton Paar, Ostfildern, Germany) was used in oscillation mode with a parallel plate configuration (plate diameter = 25 mm). The gap between the plate and the base was set at 0.5 mm. An amplitude sweep test was performed to determine the linear viscoelastic region (LVR). Afterwards, frequency sweep tests were performed at a strain amplitude of 1% (well within the LVR region) and an angular frequency range from 100 to 0.1 rad/sec. Measurements were taken at room temperature. Power law fit was used in the linear shear thinning area of the obtained rheological data to measure the shear-thinning index (n) and the consistency coefficient (k):

η = kγ^(n^−1^) (1)

where η is the viscosity, γ is the shear rate, and k is the consistency coefficient which measures the material’s resistance to flow at low rate.

### 2.7. Scanning Electron Microscopy (SEM)

The surface and cross-sections of drug loaded oral dosages were assessed using a Quanta-200 SEM microscope at 20 kV. Samples were coated under vacuum with a gold coater JFC-1200 Fine Coater (Jeol, Tokyo, Japan). In addition, photographs of tablets were acquired using a Canon EOS-1D Mark IV (Canon Ltd., Tokyo, Japan).

### 2.8. Drug Contents Using HPLC

A HPLC method was used for simultaneous detection of paracetamol and ibuprofen. Agilent 1260 HPLC system (Agilent Technologies, Waldbronn, Germany) was employed using 35:65 *v/v* mixture of 0.1% orthophosphoric acid solution (pH 2.2) and acetonitrile as a mobile phase and a Kinetex 3.5 µm XB-C18 (100 × 4.6 mm) column (Phenomenex, Aschaffenburg, Germany). An injection volume of 2.0 µL at wavelength 210 nm, column temperature of 45 °C and a flow rate of 0.5 mL/min was used. The retention times were 1.9 and 4.2 min for paracetamol and ibuprofen, respectively, and the stop time was 5 min. A calibration curve for each of paracetamol (up to 500 mg/L) and ibuprofen (up to 320 mg/L) was plotted and yielded linearity regression co-efficients of R^2^ = 0.9987 and 0.9990 with limits of detection of 22.2 and 11.2 mg/L and limits of quantification of 37.4 and 73.8 mg/L for paracetamol and ibuprofen, respectively.

### 2.9. Dissolution Test for Oral Doses

To study in vitro theophylline release for 3D printed oral dosages, an AT 7 Smart USP II dissolution test apparatus (Sotax AG, Aesch, Switzerland) was used. Each oral dose contained a dual dose of 80 mg ibuprofen and 200 mg paracetamol. A dissolution medium of 900 mL phosphate buffer BP (pH 7.2) at 37 ± 0.5 °C with a paddle speed of 50 rpm was used for 2 h. The dissolution medium was chosen according to USP 31 monograph for ibuprofen tablets [27]. Each experiment was carried out in triplicate. Samples were collected at 0, 5, 10, 15, 20, 25, 30, 35, 40, 45, 50, 60, 70, 80, 90, 100, 110, and 120 min intervals and drug concentration was determined using HPLC as specified in Section 2.8.

### 2.10. Statistical Analysis

To assess the relative influences of needle size, speed of needle movement, and extrusion multiplier, standard multiple linear regression was applied using IBM SPSS Statistics software version 25 (Chicago, IL, USA). Initial data analysis indicated no violation of the assumptions of normality, linearity, multicollinearity, and homoscedasticity.

## 3. Results and Discussion

### 3.1. Development of Embedded Phase for Pharmaceutical e-3DP

To achieve a successful e-3DP, both the embedding and embedded phases need to meet specific criteria. The embedded phase is usually required to be: (i) a shear-thinning yield stress fluid [28]; (ii) a controllable flow from the nozzle; (iii) immersible/low miscibility with embedding phase, (iv) of equal or similar density to the embedding phase; and (v) chemically compatible with the embedding material. Moreover, the embedding phase (often referred to as a matrix) should have thixotropic properties or be curable and solidify or increase in viscosity following the embedding process. Satisfying these stringent criteria is a major challenge to apply this emerging method in the pharmaceutical field.

**Figure 1:**
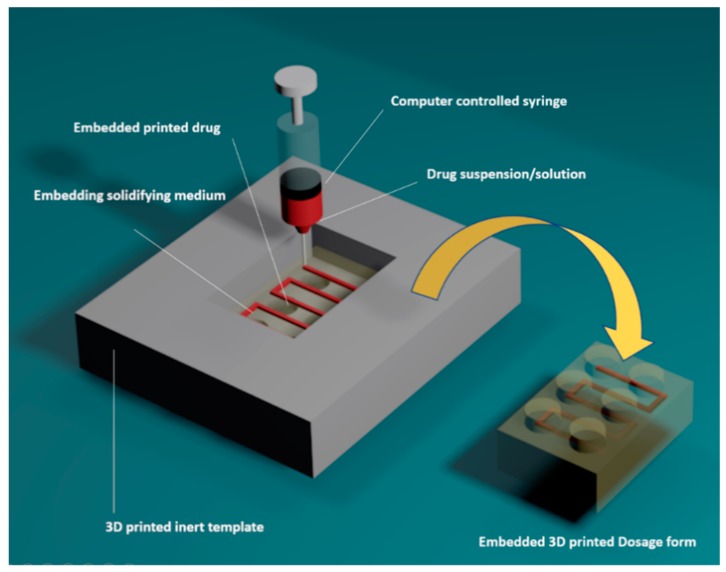
Schematic of production of embedded 3D printing of an oral dosage form using a Lego^TM^-like design as a template. Drug paste (embedded phase) is extruded in a pre-determined path into a gelatin-based matrix (embedding phase) to yield a chewable dosage form.

In this work, e-3DP enabled the encapsulation of model drugs in pre-specified shapes into an embedding material. Figure 1 illustrates the production of the proposed embedded dosage form. We adapted pharmaceutical materials to match the technical needs of e-3DP, which often requires the use of heat- or UV light-curable materials [29,30] to increase the rigidity of the embedding phase to solid or semi-solid states. Although these processes might yield a functional material, they are likely to face significant regulatory and commercial hurdles when approval is sought for oral use. Therefore, an alternative approach was tested using widely available, biodegradable, and commonly used approved excipients: gelatin and glycerol [31,32]. The combination of these excipients yields a matrix that is semi-solid at room temperature, but liquified at 75 °C when in contact with the printing plate. The inclusion of glycerol provides a sweet taste and facilitates the matrix dissolution in the gastric medium.

**Figure 2:**
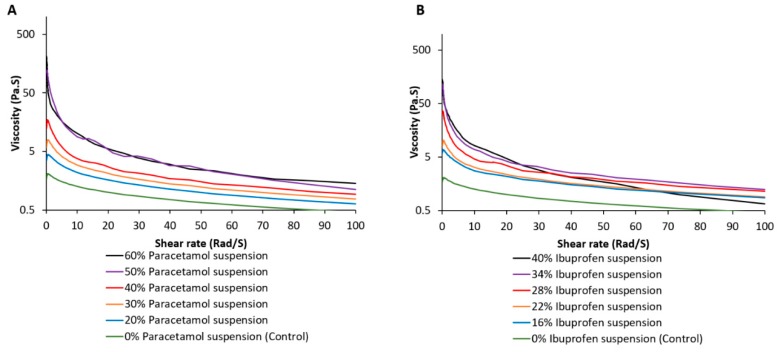
Impact of (**A**) paracetamol and (**B**) ibuprofen concentration on the rheological behavior of the applied ink (embedded phase).

Locust bean gum, a non-anionic polysaccharide biopolymer that is extracted from Carob tree seeds [33], has been used as a suspending agent for the embedded phase. The fabrication process was carefully developed to achieve a balance between the ease of flow of the embedded ink from the nozzle and the rapid solidification on/within the embedding phase upon application. To optimize this, a series of paracetamol and ibuprofen pastes (drug particle suspension in Locust bean gum solution) of increasing concentrations was prepared and the rheological behavior of these suspensions was assessed (Figure 2). In general, the viscosity of gum and suspensions decreased with increasing shear rate (Figure 2) and reflected the shear-thinning behavior of the inks used. The gum (drug-free) showed a viscosity with a consistency coefficient (k) value of 1.7235 Pa.S corresponding to blank suspensions. However, following the addition of paracetamol and ibuprofen, the k values increased reflecting the increase in viscosity of these suspensions (28% ibuprofen and 40% paracetamol, corresponding to k values of 17.022 and 10.304 Pa.S, respectively). At these drug concentrations, a consistent flow was maintained when the suspension-filled syringe was loaded onto the 3D printer. However, a further increase in the concentration of ibuprofen or paracetamol in the suspension resulted in an excessive rise in the viscosity of the suspension; therefore, rendering the suspension non-extrudable under the printing conditions. Hence, optimized concentrations of 40% paracetamol and 28% ibuprofen were used as default conditions for printing. The shear-thinning behavior of gum has been reported in previous studies and is linked to the entanglement of the polymeric chains in the solution [34]. This observation was supported by the results of the rheological study carried on the solutions containing either ibuprofen or paracetamol. This rheological behavior can be attributed to the β-d-mannose backbone with (1,6) linked α-d-galactose substitution, where mannose units within the structures have been proposed to allow self-assembly.

### 3.2. Single- and Multi-Stage e-3DP

**Figure 3:**
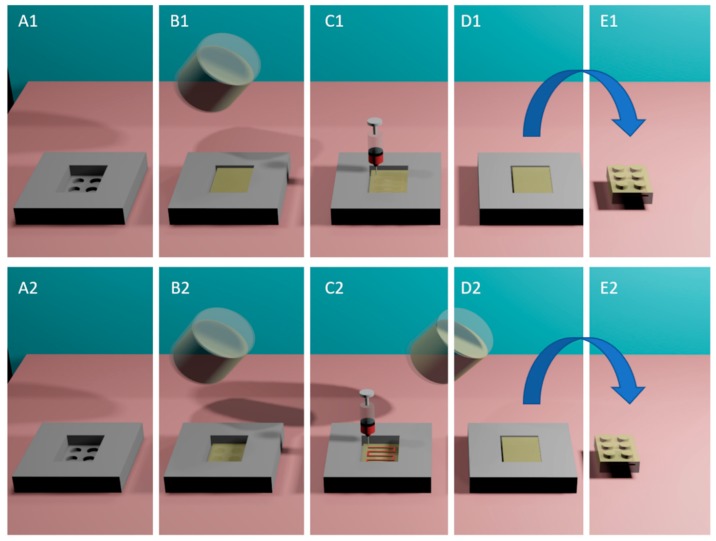
e-3DP process was carried out using (i) Single-stage printing: a 3D printed template (**A1**) is initially filled with gelatin-based matrix liquid at 70 °C (**B1**) and the drug paste is extruded instantly into the the liquified matrix (**C1**). The template is then cooled to room temperature (**D1**), and (**E1**) oral dosage form is extracted from the template; or (ii) Two stage printing: (**A2**) template is initially filled with gelatin-based matrix liquid at 70 °C and (**B2**) the template is cooled to room temperature, (**C2**) the drug paste is extruded onto the surface of the solidified gelatin-based matrix and (**D2**) the secondary layer of gelatin matrix is cast on the template surface, (**E2**) following cooling to room temperature, the dosage form is extracted.

Printing patterns were specifically designed to be included within gelatin-based Lego™-like bricks (Figure 1). The printing pattern is composed of a series of repeated parallel lines and right angles to create sufficient space between the paralleled lines to be filled with gelatin. The design was proposed to fit within 3 mm margins in the gelatin bricks. Initially, a single stage e-3DP was assessed (Figure 3A). However, this resulted in the formation of irregular lines and deviation from the pre-defined path (Supplementary data, Figure S2) as well as unreproducible printing patterns in each product (Supplementary data, Figure S3).

**Figure 4:**
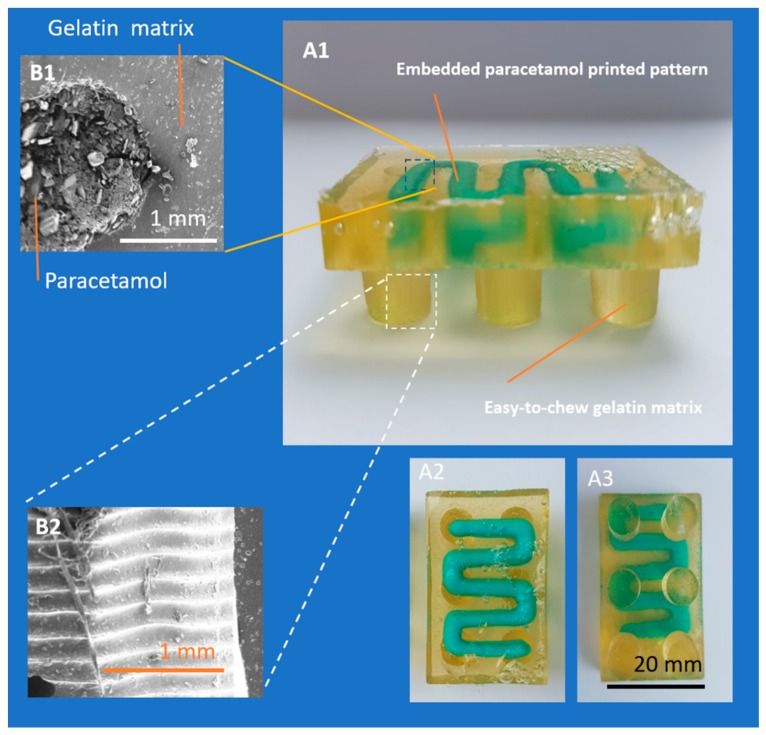
Photographs of (**A1**) side, (**A2**) front, and (**A3**) back view of Lego™-like soft gelatin with embedded paracetamol dose. SEM images of (**B1**) cross-section embedding and embedded matrix and (**B2**) surface of gelatin-based matrix.

In order to generate a more reproducible extrusion that mimics the printing patterns as per CAD design, an alternative multi-stage printing method was devised (Section 2.4), where the embedded phase (ink) is extruded on the surface of an initial layer of gelatin that is cast prior to printing. The dosage form is completed by applying a secondary layer of gelatin (Figure 3B) to fully seal the embedded phase within the gelatin matrix. This method yielded an improved printing path (Figure 4). An explanation for such improvement might a reduced resistance of the ink flow from the nozzle into atmosphere air (compared to gelatin solution) which prevented irregularity in the final printed drug patterns. Several printing parameters were assessed to define the optimized printing parameters with least variability in dosing.

### 3.3. Impact of Printing Parameters on Drug Dosing Amount and Accuracy

**Figure 5:**
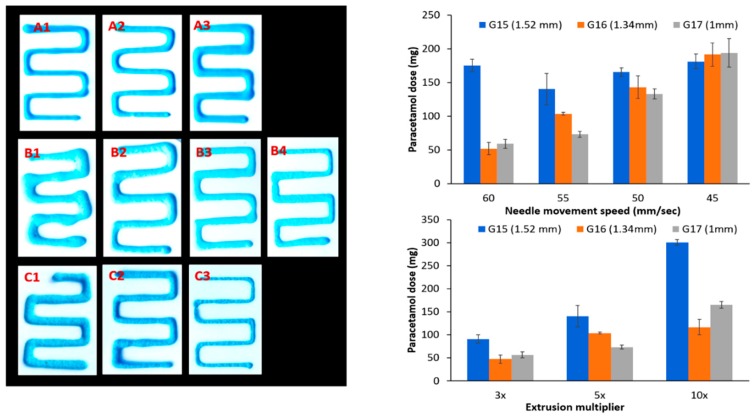
Impact of process setting on printing pattern of paracetamol paste (embedded phase): extrusion multiplier (**A1**) 3, (**A2**) 5, and (**A3**) 10. Speed of needle head: (**B1**) 45, (**B2**) 50, (**B3**) 55, and (**B4**) 60 mm/min. Needle size (**C1**) G15, (**C2**) G16, and (**C3**) G17.

The impact of the speed of movement of the printer nozzle is highlighted in Figure 5 and Table 1, where reducing the speed of printing generally resulted in an increase in the amount of dispensed paracetamol using the same printing patterns. This might be a result of extrusion of a larger amount of material (at the same flow rate) with a longer time available to complete the shape. When a larger needle size was used, it resulted in extrusion of higher doses with reduced resistance from the increased internal diameter of the needle.

One important factor in dose dispensing is the extruder multiplier (an empirical value that is proportional to the extrusion rate), which appeared to determine the doses dispensed for each needle size (Table 1). It is essential to co-ordinate these process parameters to achieve successful printing. For instance, a fast-moving needle with a limited extrusion rate will result in incomplete and voided structure, while a slow moving needle with an exaggerated extrusion rate will result in an overfilled lump. An optimized extrusion multiplier of 5×, a needle size of G16 (internal diameter of 1.3 mm) and a needle speed of 55 mm/sec yielded the most reproducible printing patterns with the narrowest standard deviation (SD% = 2%). Hence, these parameters were selected as a default setting for further testing. Under these settings, reproducible doses of 116.6 mg and 107.4 mg of paracetamol and ibuprofen, respectively, were achieved.

To quantify the relative impact of these different printing parameters, standard multi-linear regression was used and yielded the following equation:

Dispensed dose (mg) = 267.36 + 17.4 ExM − 6.88 V + 105.4 Ø(2)

where ExM is extrusion multiplier, V is the speed of needle movement (mm/sec), and Ø is the inner diameter of needle (mm). The equation highlights that dispensed dose is increased by a larger extrusion multiplier and needle size and is reduced by a faster needle speed. The multiple regression model indicated that extrusion multiplier (standardized coefficient β = 0.572, *p* < 0.002) and needle speed (β = −0.499, *p* < 0.005) are the most influential factors, followed by needle size (β = −0.374, *p* < 0.05). The model was able to describe 67.6% of the variance (F (3, 17) = 11.824, *p* < 0.0005). Other process factors such as retraction amount and speed may also influence the dispensed dose.

**Figure 6:**
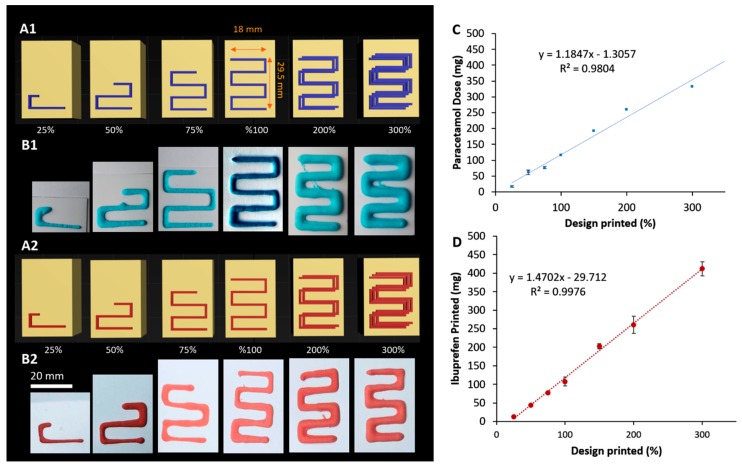
Rendered [(**A1**) and (**A2**)] and photograph [(**B1**) and (**B2**)] images of printing patterns for paracetamol and ibuprofen. Linearity between the printing pattern percentages and achieved doses for (**C**) paracetamol and (**D**) ibuprofen, respectively.

In order to establish the ability of the system to produce a different range of doses, the printing pattern was clipped or duplicated at different proportions (Figure 6). For instance, to achieve smaller doses, printing patterns were trimmed reduced to 25%, 50%, or 75% to cover dose ranges of 16–77 mg and 12–76 mg of paracetamol and ibuprofen, respectively. To achieve higher doses, the printing design was repeated following the same printing path twice or three times followed by an elevation of height at 0.5 mm after each layer (Table 2). Figure 6 shows rendered images and photographs of the printed patterns using this approach for both paracetamol and ibuprofen. For both model drugs, good linearity [R^2^ = 0.9804 (paracetamol) and 0.9976 (ibuprofen)] was achieved between the percentage of printed patterns and the achieved doses. The negative intercept values in the trendline equations suggest that there is a reduced extruded ink in small size designs, e.g., at 25% of design pattern. This could be a direct result of the shear-thinning behavior of the ink, where dynamic viscosity is high at the beginning of the printing process before it is reduced as ink flow continues. Another source of variation in the dispensed dose could result from resistance to ink flow by the layer already extruded from the needle nozzle while printing the next layer. This could be mitigated by careful adjustment of the height that the needle travels on *z*-axis following deposition of each layer. For this technology to be adopted in a clinical setting, the precision of dosing needs to be improved. This could be achieved by enhancing the retraction mechanism in both nozzle design and software.

The system enables different printing patterns for dose titration without the need for altering the concentration of the embedded phase or changing the formulation or the volume of the external embedding phase. Such a challenge is often faced when relatively large drug loading needs to be incorporated in gel structure.

### 3.4. Drug Release from 3D Printed Oral Doses

**Figure 7:**
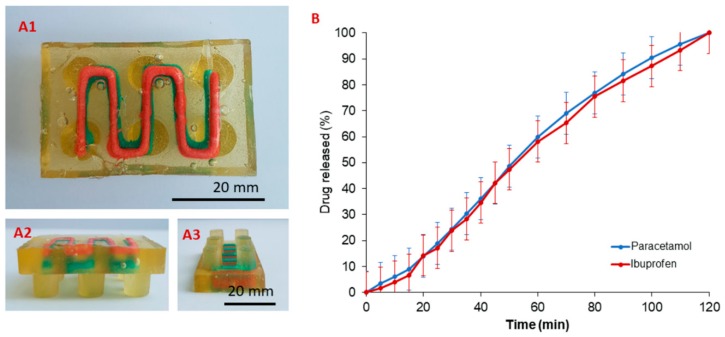
(**A1**) Bottom and [(**A2**) and (**A3**)] side views of oral concept gelatin-based dosage form for paracetamol (blue) and ibuprofen (red). (**B**) Drug release from the dosage form in simulated intestinal fluids (pH 7.2) using USP II dissolution test (*n* = 3, 50 rpm, error bar represents standard deviation).

Another important aspect for an age-appropriate dosage form is the ability to accommodate different drugs in a minimal number of doses. Paracetamol and ibuprofen have frequently been used in combination for management of numerous pediatric conditions such as fever, post-operative pain, and pain relief [35,36,37,38]. The concept described herein has potential clinical utility under circumstances when combined rather than alternate therapy is required. The ability of the technology to easily produce drug combinations at variable ratios in a single oral dosage form is of great interest. In addition, reducing the number of individual medicines into a single dosage form is appealing to families, minimizing the need to adhere to several drug administrations which can lead to missed, omitted, or dual doses. Figure 7 demonstrates ibuprofen and paracetamol release from gelatin Lego™-like bricks. The pH of the dissolution medium was selected as recommended by USP pharmacopial monograph for ibuprofen and was within the reported range of salivary pH in healthy individuals [39]. Upon introduction in dissolution medium, the gelatin-based matrix will dissolve quickly, leaving a paste of printed drug patterns to dissolve at a slower rate.

It is interesting to highlight that both drugs were released at similar rate despite the relatively higher solubility of paracetamol compared to ibuprofen at intestinal pH (approximately 20.9 versus 3.9 g/L, respectively) [40,41]. This may be due to the slow dissolution of locust bean gum, a galactomanon that acts as a gel former in the embedded phase. It is likely that the release patterns of both drugs were governed by drug diffusion through diffusing spaces within the network of macromolecular chains [42,43]. In the future, it will be possible to manipulate drug release by applying different ‘ink’ matrixes to accommodate for different release patterns.

One potential advantage of applying e-3DP for production of chewable products is the possibility of encapsulation of drug paste in an acceptable gelatin matrix. Although some of the embedded materials are likely to be in contact with taste buds in the tongue, the system can potentially reduce the extent and the duration of such interaction, hence providing some advantage for bitter drugs. Another important novelty aspect in this concept dosage form is its modular nature that can include individualized drugs (single or combination) and doses in a single chewable form, hence offering the possibility for more complex dosing regimens and high levels of individualization. For example, the possibility of titrating the relative doses of paracetamol and ibuprofen in a single dosage form is an advancement on the current options available for circumstances in which combination therapy is indicated.

## 4. Conclusions

We have demonstrated the potential of using e-3DP for the delivery of bespoke concept chewable soft dosage forms for use in different age groups including children. The soft nature and sweet taste of the matrix may help patients with swallowing difficulties or palatability challenges. Compared to traditional oral disintegrating tablets and soft gels, the method allows independent design of both shell and core materials without modifying the external embedded matrix composition. In addition, by manipulating the geometry of printing patterns, simultaneous dosing and drug release of two different active pharmaceutical ingredients was achieved. Moving forward, the use of other liquid inks as an embedding phase (for example, hydrogels, suspension of nanoparticles, therapeutic biologics) offers the possibility of extending e-3DP printing to plethora of patient-specific oral drug delivery systems.

## Figures and Tables

**Table 1:** Impact of needle size, extrusion multiplier, and speed of the needle movement on the dose paracetamol in mg (100% of printing pattern).

Extrusion Multiplier *	Needle Size G15 (1.52 mm) ±SD	SD%	Needle Size G16 (1.34 mm) ±SD	SD%	Needle Size G17 (1 mm) ±SD	SD%
3×	90.8 ± 4.3	4.7%	47.4 ± 4.2	5.1%	56.5 ± 6.9	12.2%
5×	140.3 ± 23.3	16.6%	103.6 ± 2.1	2%	73.3 ± 4.4	6%
10×	300.8 ± 9.4	3.12%	116.6 ± 7.2	6.2%	165.2 ± 22.4	13.6%
Speed (mm/sec) **						
60	175.5 ± 9.0	5.1%	51.8 ± 9.1	17.6%	59.2 ± 6.7	11.3%
55	140.3 ± 23.3	16.6%	103.6 ± 2.1	2%	73.3 ± 4.4	6%
50	165.8 ± 6.2	3.7%	143.2 ± 16.9	11.8%	133.0 ± 7.3	5.5%
45	181.4 ± 11.0	6.1%	191.5 ± 17.2	9%	194.0 ± 21.3	10.9%

* The speed of the nozzle movement was fixed at 55 mm/sec. ** Extrusion multiplier was fixed at 5×.

**Table 2:** Dosing of paracetamol and ibuprofen using different percentages of the printing pattern.

Printing Pattern	Paracetamol Dose (mg) ±SD	SD%	Ibuprofen Dose (mg) ± SD	SD%
25%	16.1 ± 2.7	16.9%	12.1 ± 1.0	8.1
50%	61.7 ± 5.1	8.2%	44 ± 1.9	4.4
75%	77 ± 12.2	15.8%	77 ± 4.0	5.2
100%	116.7 ± 7.0	6.0%	107.4 ± 12.1	11.3
200%	259.4 ± 16.8	6.5%	260.3 ± 23.6	9.1
300%	329.8 ± 6.4	2%	411.9 ± 19.2	4. 7

## Supplementary Materials

The following are available online at https://www.mdpi.com/1999-4923/11/12/630/s1. Figure S1. Modification of dual FDM 3D printer to accommodate a liquid/semisolids dispenser (right) in combination with FDM 3D printer head (left). Figure S2. Soft dosage forms with paracetamol paste inside (blue line) fabricated via one-step e-3DP with different printing speeds: (A) 50, (B) 60, (C) 65, and (D) 70 mm/min. Figure S3. Soft dosage forms with paracetamol inside (blue line) fabricated via one-step e-3DP with printing speed of 55.0 mm/min.
