# Supplementary material for: Embedded 3D Printing of Novel Bespoke Soft Dosage Form Concept for Pediatrics
Source: Pharmaceutics. 2019 Nov 26;11(12):630. doi: 10.3390/pharmaceutics11120630 (PMC6956071; doi:10.3390/pharmaceutics11120630)
Supplement: Supplementary file 1 [file pharmaceutics-11-00630-s001.pdf]

pharmaceutics

Supplementary Materials: Embedded 3D Printing of Novel Bespoke Soft Dosage Form Concept for Paediatrics

Katarzyna Rycerz, Krzysztof Adam Stepien, Marta Czapiewska, Basel T. Arafat,
Rober Habashy, Abdullah Isreb, Matthew Peak and Mohamed A. Alhnan

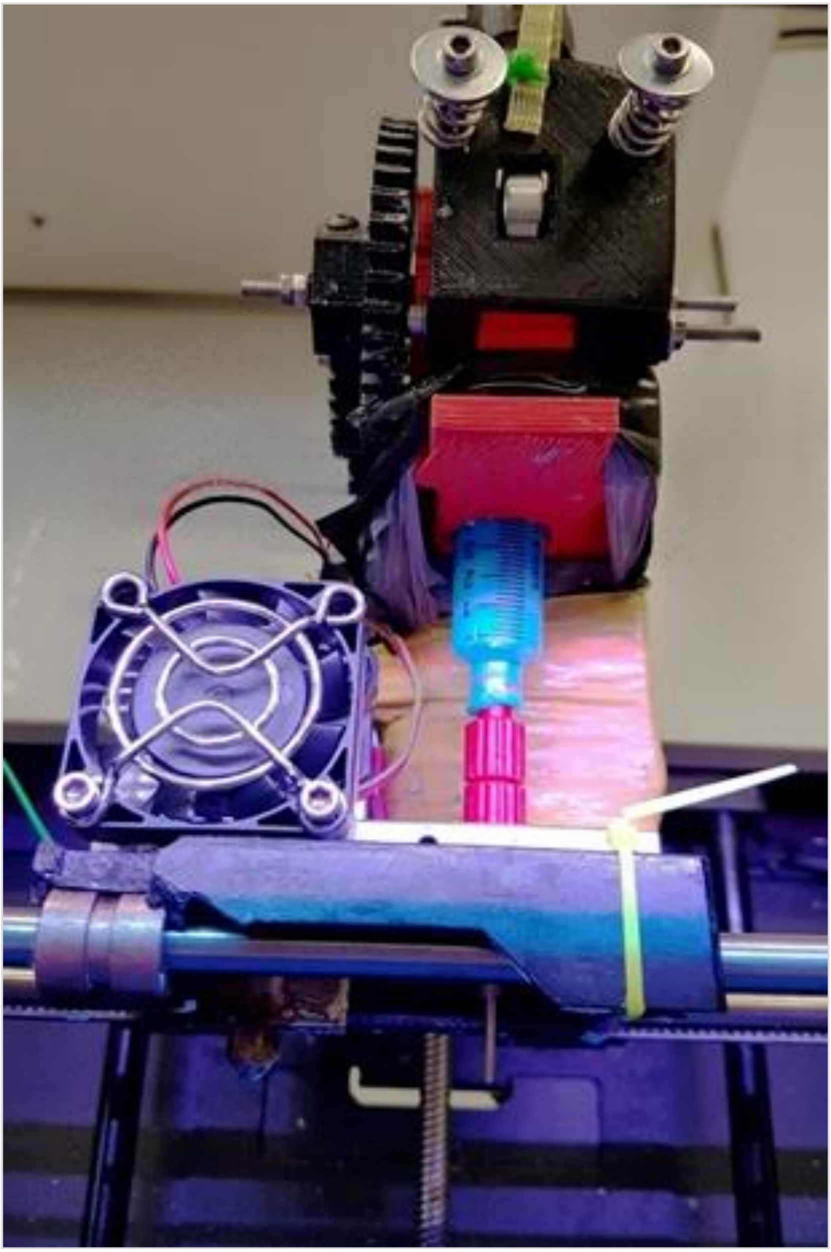

Figure S1. Modification of dual FDM 3D printer to accommodate a liquid/semisolids dispenser (right) in combination with FDM 3D printer head (left).

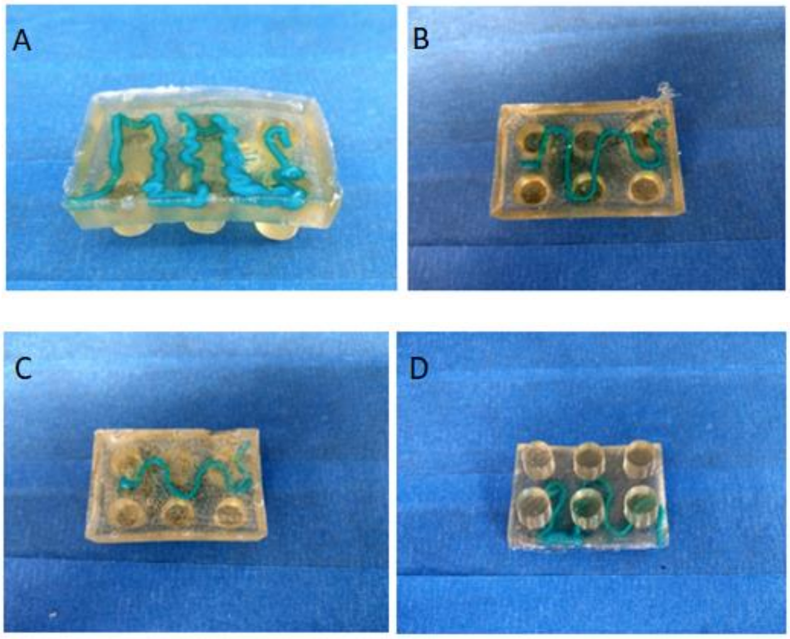

Figure S2. Soft dosage forms with paracetamol paste inside (blue line) fabricated via one-step e-3DP with different printing speed: (**A**) 50, (**B**) 60, (**C**) 65, (**D**) 70 mm/min.

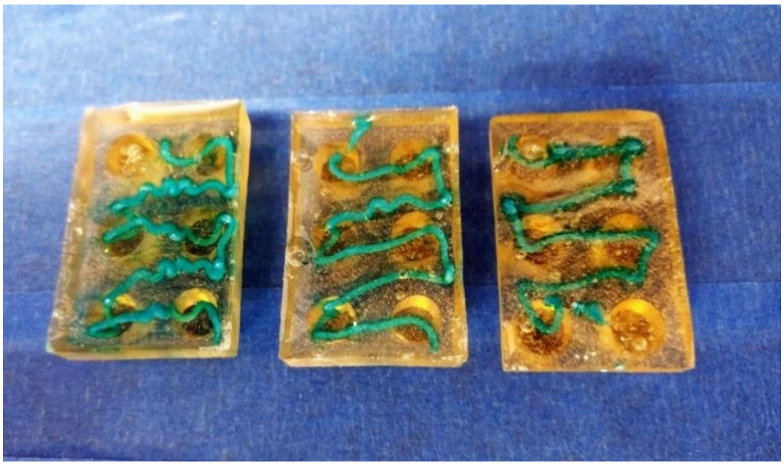

Figure S3. Soft dosage forms with paracetamol inside (blue line) fabricated via one-step e-3DP with printing speed 55.0mm/min.
